# The effect of palmitate supplementation on gene expression profile in proliferating myoblasts

**DOI:** 10.1007/s10565-016-9324-2

**Published:** 2016-04-25

**Authors:** K. Grabiec, A. Majewska, Z. Wicik, M. Milewska, M. Błaszczyk, K. Grzelkowska-Kowalczyk

**Affiliations:** Department of Physiological Sciences, Faculty of Veterinary Medicine, Warsaw University of Life Sciences (SGGW), Nowoursynowska 159, 02-776 Warsaw, Poland

**Keywords:** Cytokines, Growth factors, Long-chain fatty acids, Myogenesis, Signaling, Transcriptome

## Abstract

High-fat diet, exposure to saturated fatty acids, or the presence of adipocytes in myoblast microenvironment affects skeletal muscle growth and function. The aim of the present study was to investigate the effect of palmitate supplementation on transcriptomic profile of mouse C2C12 myoblasts. Global gene expression was evaluated using whole mouse genome oligonucleotide microarrays, and the results were validated through qPCR. A total of 4047 genes were identified as differentially expressed, including 3492 downregulated and 555 upregulated genes, during a 48-h exposure to palmitate (0.1 mmol/l). Functional classification showed the involvement of these genes in several processes which regulate cell growth. In conclusion, the addition of palmitate modifies the expression of genes associated with (1) myoblast responsiveness to hormones and growth factors, (2) cytokine and growth factor expression, and (3) regulation of cell-cell and cell-matrix communication. Such alterations can affect myoblast growth and differentiation; however, further studies in this field are required.

## Introduction

Skeletal muscle is a key insulin-responsive tissue, which is important for glucose and fatty acid utilization (Houmard 2008). The fetal stage is crucial for skeletal muscle development in humans and large farm animals because there is no increase in muscle fiber number after birth (Zhu et al. 2006). The skeletal muscle mass is, therefore, closely related to the number of cells which trigger the myogenic program and are able to undergo hypertrophy at later stages of muscle development (Bonnet et al. 2010).

According to a recent study on human and animal models, secondary myogenesis that mainly accounts for muscle mass is susceptible to the availability of nutrients and humoral factors and, thus, suboptimal fetal environments decrease the number of myofibers. Recent studies revealed that maternal high-fat feeding and obesity increase fat-to-lean mass ratio and cause impaired glucose tolerance in offspring (reviewed in Warner and Ozanne 2010). Humoral factors associated with maternal obesity can reduce fetal muscle fiber number and/or diameter and promote adipogenesis and fibrogenesis (Du et al. 2010). Downregulation of myogenesis and Wnt/β-catenin signaling, probably resulting from activation of inflammatory nuclear factor κB (NF-κB) pathway in fetal skeletal muscle, has been reported in an obese pregnant sheep model (Tong et al. 2009). Extracellular factors acting during myogenesis control myoblast proliferation and differentiation through regulation of signaling pathways for the expression of specific transcription factors and morphological changes (Le Grand and Rudnicki 2007). According to the cell niche concept, myoblast behavior is determined by the stimuli acting in the surrounding environment (Ohlstein et al. 2004), in which adipocyte-derived fatty acids can be important signaling molecules. These influences may, in turn, modify the expression and secretion of factors which regulate numerous biological processes in autocrine, paracrine, and endocrine manner (Henningsen et al. 2010; Chan et al. 2011). Alterations in muscle cell secretome during myogenic differentiation support an important and active contribution of myoblasts in regulation of their microenvironment (Griffin et al. 2010; Ge et al. 2013).

Microarray-based profiling enables the simultaneous measurement of the expression of multiple genes and the identification of genes and proteins that contribute to the regulation of cellular processes. In the present study we used a microarray technique for the comprehensive analysis of palmitate effect in mouse C2C12 myoblasts. Global gene expression was evaluated using whole mouse genome oligonucleotide microarrays, and the results were validated through real-time PCR. Gene ontology analysis was used to classify gene functions. We demonstrated that palmitate regulates genes involved in different pathways associated with myoblast growth and development, i.e., signal transduction, cell commitment, differentiation, cell-cell and cell-matrix signaling, and calcium homeostasis. These results can provide a basis for future studies to investigate specific mediators of effects of fatty acids on skeletal muscle growth.

## Materials and methods

### Preparing fatty acid-containing media

The solution of palmitate for the experimental treatment was prepared by mixing with FFA-free bovine serum albumin, as described by Coll et al. (2008). First, palmitate (the sodium salt, obtained from Sigma-Aldrich), was dissolved in ethanol and then diluted 1:100 in Dulbecco modified Eagle’s medium (DMEM), supplemented with 2 % (*w*/*v*) fatty-acid free bovine serum albumin. Palmitate complexed to BSA was added to the experimental medium to obtain the final concentration of 0.1 mmol/l. This concentration of palmitate does not mimic hyperlipidemic conditions; however, it does not exert the toxic effect.

### Cell culture

A research work was carried out on the murine myogenic C2C12 cell line (satellite cells from thigh muscle), purchased from the European Collection of Animal Cell Culture (ECACC). This cell line undergoes proliferation and differentiation in response to growth factors present in the extracellular environment; thus, it is a useful model to study the mechanisms controlling myogenesis (Yaffe and Saxel 1977).

Mouse C2C12 myoblasts were maintained free of contamination at the exponential phase of growth in DMEM supplemented with 10 % fetal bovine serum (FBS) and an antibiotic-antimycotic mixture (Life Technologies), in controlled humidified air supplemented with 5 % CO_2_, at 37 °C. The growing medium was changed every other day. After reaching ~40 % confluence, the proliferating myoblasts were subjected to 48-h exposure to palmitate (0.1 mmol/l) added to 5 % FBS/DMEM, according to the experimental protocol described previously (Lee et al. 2009). The control cultures were maintained in 5 % FBS/DMEM, containing the same amounts of ethanol and FFA-free bovine serum albumin, as the experimental medium.

### Analysis of myoblast number

In order to verify the effect of palmitate supplementation on cell number, C2C12 myoblasts were seeded onto Petri dishes and cultured as described above. Changes in cell number were monitored using a phase contrast microscope (IX 70, Olympus Optical Co., Hamburg, Germany) and a Kodak DC 290 zoom digital camera (Eastman Kodak Co., Rochester, NY, USA). The images of 10 independent visual fields for each dish were captured before (0 h) and after the 48-h treatment, and myoblast number in control and palmitate-treated cultures were compared using the images related to the same visual field. The results were presented as the percentage of initial cell number.

### Assessment of DNA content and cell respiration

A crystal violet (CV) assay was performed to determine the total amount of nuclear DNA. The cells were cultured in 96-well plates and fixed with 75 and 100 % methanol for 20 min, and the monolayer was stained using the crystal violet solution (2 mg/ml) for 5 min. The excess unbound dye was removed by washing the plates with water. The bound crystal violet was released after adding 1 % SDS for 30 min.

Viability of proliferating cells was determined using 3-(4,5-dimethylthiazol-2-yl)-2,5-diphenyltetrazolium bromide (MTT) assay, as described previously (Jacobson et al. 1994) with minor modifications. The cells were seeded in 96-well plates, and after incubation time, the MTT solution (0.5 mg/ml) in PBS was added to each well. The plates were then incubated for 4 h at 37 °C. The reaction product, i.e., precipitated formazan, was solubilized in 100 % DMSO (100 μl/well).

In both assays, the absorbance was measured on the Infinite 200 PRO Tecan^™^ multidetection microplate reader (Tecan, Mannedorf, Switzerland) at a wavelength of 570 nm.

### Microarray analysis

Total RNA was extracted and purified from the C2C12 cells using the Total RNA kit (A&A Biotechnology, Poland) according to the manufacturer’s protocol. The isolated RNA samples were dissolved in RNase-free water. The quantity of the isolated RNA was measured using NanoDrop (NanoDrop Technologies, USA). The RNA samples were treated with DNase I to eliminate DNA contamination and were subsequently purified using the RNeasy MiniElute Cleanup Kit (Qiagen, Germany). Subsequently, the RNA samples were analyzed on a BioAnalyzer (Agilent, USA) to measure the final RNA quality and integrity. Total RNA (1000 ng) from each sample was amplified and labeled using the Quick Amp Labeling Kit (Agilent, USA) according to the protocol for Agilent Gene Expression oligo microarrays (version 5.7, March 2008). The Agilent Whole Mouse Gene Expression (Mouse GE 4 × 44K version 2) oligonucleotide microarray slide (containing four microarrays; array ID 026655) was used for the hybridization. Each microarray contained 39,485 oligonucleotide probes that correspond with 27,122 of RefSeq (Entrez) gene counts. The complementary RNA (cRNA) from one control sample (labeled with cyanine-3) and one experimental sample (labeled with cyanine-5) were hybridized onto each microarray, resulting in the analysis of RNAs from four control and four experimental cultures. The hybridized microarrays were scanned on an Agilent G2505C microarray scanner. Agilent Feature Extraction (FE) software version 10.7.3.1 was used to extract data, place microarray grids, reject outlier pixels, accurately determine feature intensities and ratios, flag outlier pixels, and calculate statistical confidences. The statistical analysis was performed using the Gene Spring 12 software (Agilent, USA). The raw data were preprocessed to remove variability across and within array samples. To minimize the non-biological variability across arrays, the raw data were first log2 transformed. Total detected entities were filtered by flags (detected, non-detected) and error (coefficient of variation: CV <50.0 %) to remove very low signal entities and to select reproducible signal values of entities among the replicated experiments.

### Quantitative PCR

To validate the microarray data, the expression of four genes (*Arpc3*, *Tfpi*, *Usp47*, *Angptl4*) was analyzed using quantitative real-time PCR. The messenger RNA (mRNA) gene sequences were obtained from the National Center for Biotechnology Information (NCBI) database, and the primers were designed using the PRIMER3 software (free online access) and validated using Oligo Calculator (free online access) and Primer-Blast (NCBI database). The primer sequences are listed in Table 1. The *Rpl13a* gene was used as a reference gene for the normalization of target gene expression. The expression stability value of the housekeeping gene *Rpl13a* was determined using the geNorm and NormFinder programs. The *M* value equals 0.514 and the standard deviation equals 0.459.

**Table 1:** The primers used for qPCR

Gene symbol	Primer sequence	Optimum annealing temp. (°C)	Source
*Arpc3* (NM_019824)	Fr: AACAGGAGGACGAGATGATG	58	Designed
	Rv: CACCACTTGCTGGGTTTATC		
*Tfpi* (NM_011576.1)	Fr: AATTTTGGGACCTCATCTCC	58	Designed
	Rv: AACTCGGGAACAAGGCTAAG		
*Usp47* (NM_133758.3)	Fr: TGGATCACACCAGTGACAAG	58	Designed
	Rv: GCAGTGGACCTATGAACCTG		
*Angptl4* (NM_020581)	Fr: CTTTCCCTGCCCTTCTCTACT	58	Designed
	Rv: ATGGCTACAGGTACCAAACCA		
*Rpl13a*	Fr: AATGTGGAGAAGAAAATCTGCAA	58	Designed
	Rv: TCATTTTCAACACTGAAGCTCAA		

The primers were designed using the PRIMER3 software (free online access) and checked using the Oligo Calculator (free online access) and the Primer-Blast (NCBI database). The *RPl13a* gene was used as a non-regulated reference gene for normalization of the target gene expression

The total RNA was reverse transcribed to first strand complementary DNA (cDNA) using the High Capacity cDNA Reverse Transcription Kit (Applied Biosystems, USA), following the manufacturer’s protocol. RT-PCR was performed on a Stratagene Mx3005P quantitative PCR instrument using the SYBR Select Master Mix (Applied Biosystems, USA).

The qPCR program included preincubation step at 50 °C for 2 min and 95 °C for 2 min, and it was continued with 40 cycles consisting of a denaturing step at 95 °C for 15 s, an annealing step at 58 °C for 15 s, and an elongation step at 72 °C for 30 s. All PCR reaction efficiencies were between 92 and 105 %, and the product melting curves showed single products and the absence of a product in the negative controls.

The results were calculated using the 2^−ΔΔ*C*T^ method (Livak and Schmittgen 2001). The expression changes are shown as the relative upregulation or downregulation normalized to the internal reference gene, *Rpl13a*. The experiment was performed three times.

### Statistical analyses

The results of the MTT and CV tests are representative of four separate experiments performed in triplicate (*n* = 12). The results were presented as means ± SE. For each assay, the Student *t* test was used for the comparison of two means (control vs experimental treatment) and the criterion for statistical significance was *P* < 0.05. The analyses were performed using GraphPad Prism 5 (GraphPad Software, USA).

Differential gene expression was statistically analyzed using an unpaired *t* test to examine the null hypothesis of no differential expression between the control and experimental groups. The statistical significance was set at *P* ≤ 0.05. Moreover, the Benjamini-Hochberg false discovery rate (FDR) test, with a ≤0.05 significance level, was employed. All significant changes with fold-change absolute values greater than or equal to 1.6 were selected for ontological analyses. The data obtained from these analyses have been deposited in the NCBI Gene Expression Omnibus and are accessible via GEO Series accession number GSE75378. All genes significantly altered in the analyses presented herein were analyzed using commercial Pathway Studio software (Agilent, USA). The Agilent microarray technology used in this study is one of the most accurate, where the minimum detectable fold change (MDFC) is below 1.3 (Stafford 2008). The fold-change cutoff, in the range of 1.6–2.0, is often used in the analysis of microarray data (Morey et al. 2006; Munoz et al. 2012). This approach helped to avoid exclusion of particularly interesting genes, which may display slight expression changes associated with the performance of their important biological function, from further analyses (Lee 2001; Tarca et al. 2006). In addition, it has been dictated by the results obtained from the analysis of interactions between the analyzed genes carried in the Pathway Architect (Agilent, USA), which showed that genes with significant but low changes in expression can act as key genes in the myogenesis process. Moreover, genes with fold change equal or higher than 2 account for only 6 % of total genes with significant changes in expression after FDR correction. Genes with 1.6 fold change in expression accounted for 61 % of all genes with significant changes in expression.

## Results

### Myoblast characteristics under palmitate treatment

**Fig. 1:**
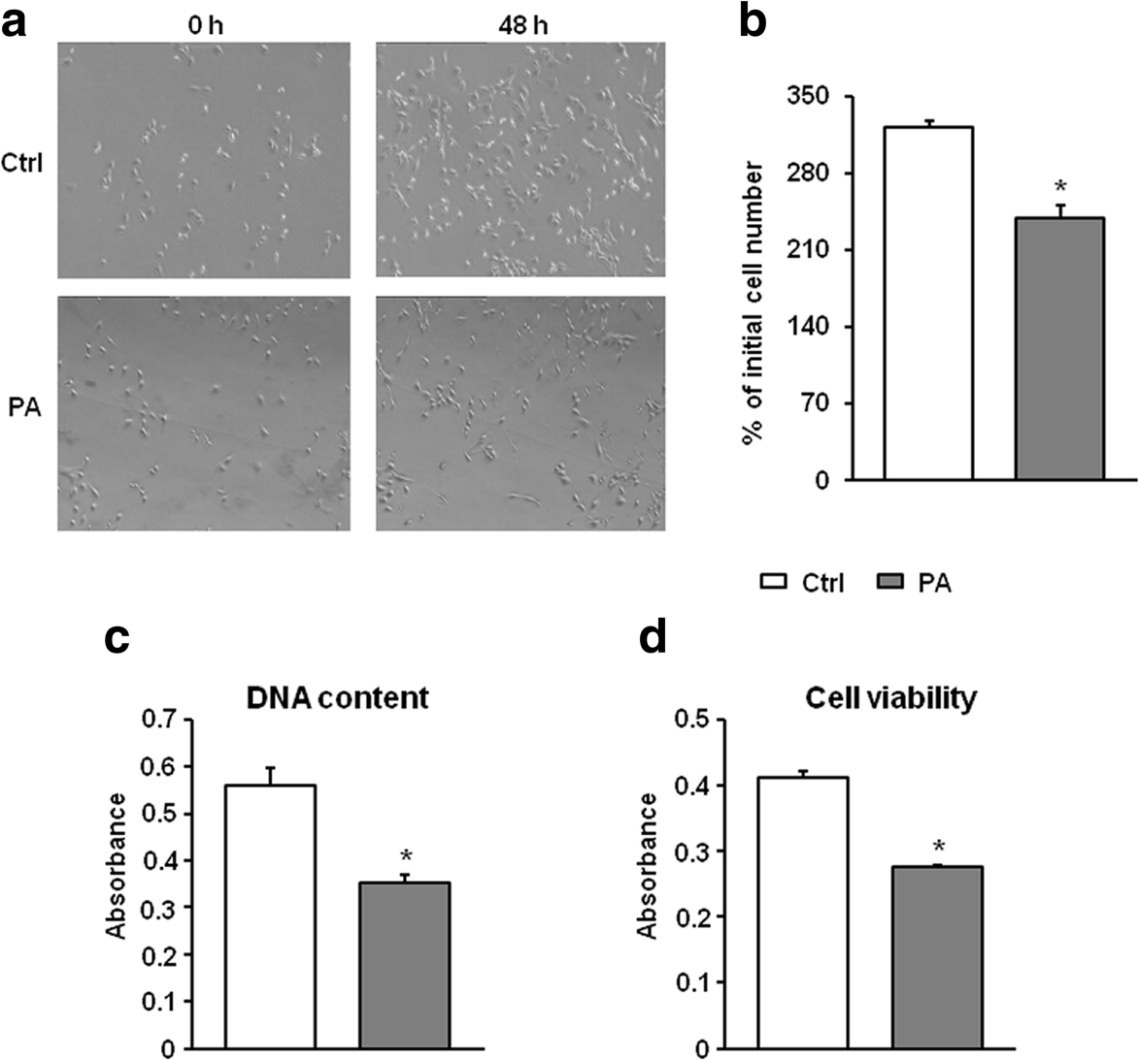
The characteristics of mouse C2C12 myoblasts used in the study. **a** The effect of 48-h palmitate supplementation on myoblast number. Representative phase-contrast images (magnification ×100) of control (*Ctrl*) and palmitate-treated (*PA*; 0.1 mmol/l) cultures. **b** The results of myoblast counting are shown as the percentage of the initial cell number, *n* = 3. **c** DNA content (crystal violet test) and **d** cell viability (MTT assay) of myoblasts exposed to palmitate for 48 h, *n* = 12. The results represent the mean ± SE, *Significantly different vs control value

The presence of palmitate (0.1 mmol/l) significantly decreased the number of myoblast in proliferating cultures after the 48-h treatment (by 25.8 % in comparison to control value, *P* = 0.004, Fig. 1). This was confirmed by the results of the crystal violet test (drop by 37.4 % vs control value, *P* < 0.0001) and lower cell viability (drop by 33.1 % below control value in MTT assay, *P* < 0.0001).

### Microarray analysis

Fold-change analysis with the absolute cutoff criteria set at 1.6 followed by *t* test and Benjamini-Hochberg FDR correction was used to identify 3492 transcripts that were downregulated and 555 transcripts that were upregulated in palmitate-treated myoblasts. A total of 4047 of these genes were annotated for *Mus musculus*.

### Validation of gene expression data by quantitative real-time PCR

To validate the microarray data, we randomly selected four genes significantly regulated by the presence of palmitate with an absolute fold change of at least 2.0 (Table 2). The real-time PCR analysis showed expression pattern changes that were similar to those observed in the microarray analysis, confirming the downregulation of *Arpc3* (actin-related protein 2/3 complex, subunit 3) and the upregulation of *Usp47* (ubiquitin-specific peptidase 47), *Tfpi* (tissue factor pathway inhibitor), and *Angtpl4* (angiopoietin-like 4).

**Table 2:** The expression of randomly selected genes assessed by microarray analysis and real-time qPCR in mouse C2C12 myoblasts treated for 48 h with palmitate (0.1 mmol/l)

Gene name	cDNA microarray	Real-time qPCR
*Arpc3*	−3.9	0.8 ± 0.09*
*Tfpi*	2.1	1.59 ± 0.20*
*Usp47*	2.0	1.26 ± 0.15*
*Angptl*4	2.8	1.93 ± 0.25*

The expression changes are shown as relative up- or downregulation normalized by the internal reference gene (*Rpl13a*). The data from three separate experiments were used to calculate the means ± SE

*Significantly different vs control value (*P* < 0.05)

In order to verify potential direct effects of palmitate on gene expression, the mRNA levels of these genes were measured at shorter time points (2, 6, 12, and 24 h, Table 3). The presence of palmitate caused an increase in *Angtpl4* mRNA within a few hours. The expressions of other genes were modified after either 12 (*Usp47*) or 24 h (*Arpc3* and *Tfpi*). Moreover, the effects of palmitate supplementation on *Tfpi* and *Usp47* after 24 (Table 3) and 48 h of treatment (Table 2) were opposite.

**Table 3:** The expression of randomly selected genes assessed by real-time qPCR in mouse C2C12 myoblasts treated for 2, 6, 12, and 24 h with palmitate (0.1 mmol/l)

Gene name	Time			
	2 h	6 h	12 h	24 h
*Arpc3*	0.96 ± 0.04	0.95 ± 0.11	0.91 ± 0.05	0.55 ± 0.02*
*Tfpi*	1.21 ± 0.26	0.83 ± 0.18	0.87 ± 0.05	0.65 ± 0.02*
*Usp47*	1.1 ± 0.24	0.81 ± 0.23	0.69 ± 0.07*	0.47 ± 0.03*
*Angptl4*	2.38 ± 0.05*	2.99 ± 0.21*	3.98 ± 0.25*	2.96 ± 0.12*

The expression changes are shown as relative up- or downregulation normalized by the internal reference gene (*Rpl13a*). The data from three separate experiments were used to calculate the means ± SE

*Significantly different vs control value at the same time point (*P* < 0.05)

### Gene ontology analysis

**Fig. 2:**
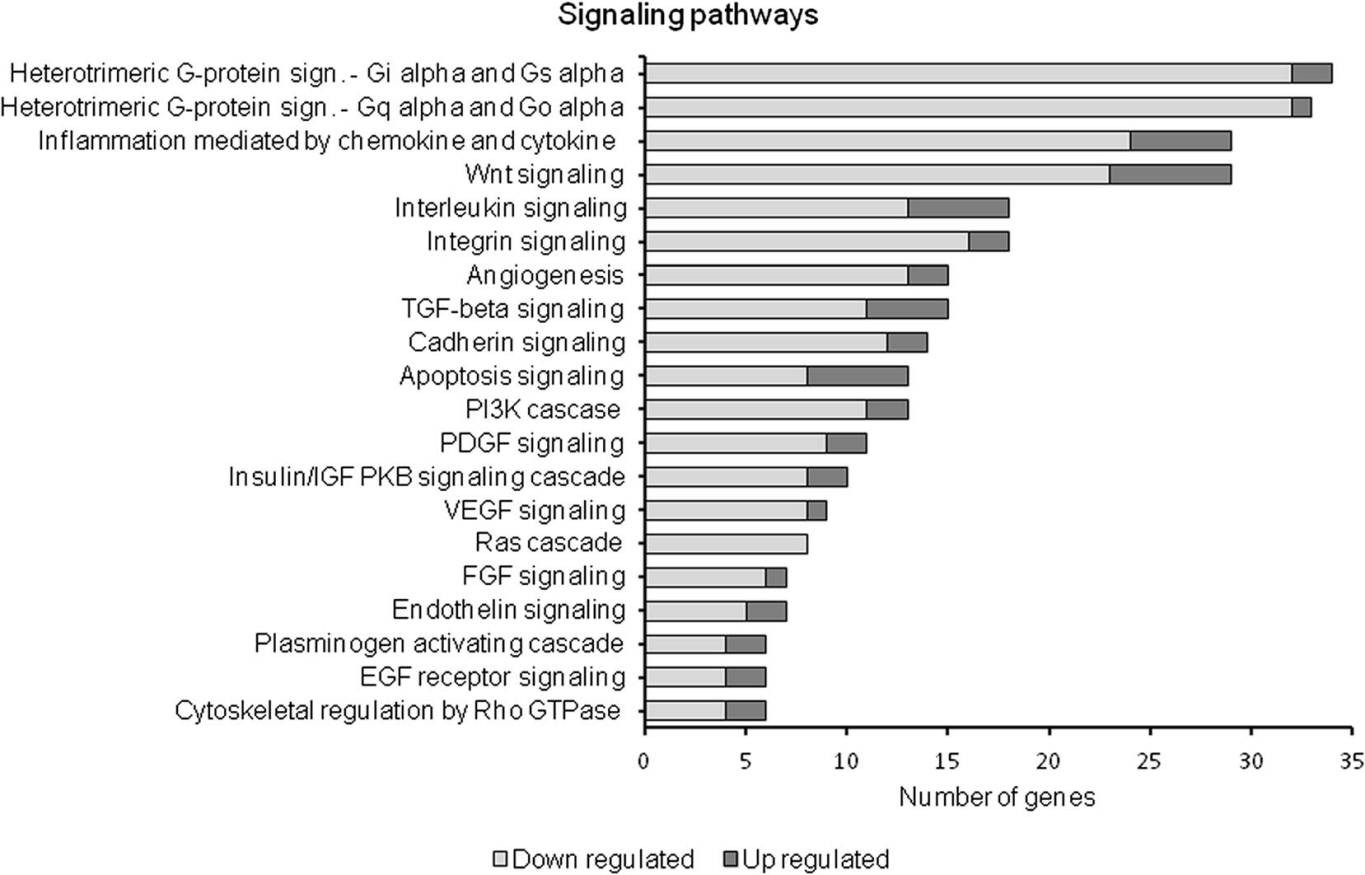
Signaling pathways enriched by differentially expressed genes in mouse C2C12 myoblasts exposed for 48 h to palmitate (PA, 0.1 mmol/l). The analysis was performed using the Panther classification system. The results were sorted by *P* value (cutoff ≤0.05) and absolute fold change (cutoff ≤1.6)

The gene ontology (GO) analysis assigns different genes to different GO categories. Table 4 shows the major GO biological processes and GO molecular function groups associated with differentially expressed genes in myoblasts exposed to palmitate. The most significant pathway in which the genes regulated in the presence of palmitate were involved was the signal transduction. The treatment of myoblasts with palmitate modified the expression of 7–18 % of genes that play roles in several biological processes associated with muscle cell proliferation and growth. The addition of palmitate altered the expression of 10–24 % of genes associated with signal transducer activity, G-protein coupled receptor, cytokine and growth factor activity, and metallopeptidase function. Numerous identified genes were involved in the metabolic process, which was not unexpected, in view of the well-documented role of saturated fatty acids in skeletal muscle metabolism. According to the Panther classification, apoptosis is beyond the “top list” of biological processes which are highly enriched in genes affected by addition of palmitate. In fact, we found “positive regulation of apoptosis,” with total entities amounting to 232, overlapping entities amounting to 21, and *P* = 0.035, indicating a rather minor role of this process in the control of myoblast number. The Panther analysis of identified genes revealed that they were involved in numerous intracellular pathways associated with cytokine/chemokine and growth factors signaling and that the majority of these genes was downregulated through experimental treatment (Fig. 2).

**Table 4:** Gene ontology groups enriched in the differentially expressed genes in mouse C2C12 myoblasts exposed to palmitate (0.1 mmol/l) for 48 h

	Total entities	Overlap	Corrected *P* value
Biological processes			
Signal transduction, GO:0007165	3586	675	0.000017
Metabolic processes, GO:0008152	2421	184	0.000589
Cell differentiation, GO:0030154	735	84	0.000226
Cell adhesion, GO:0007155	686	71	0.000413
Proteolysis, GO:0006508	657	62	0.000597
Inflammatory response, GO:0006954	316	39	0.000492
Cell-cell signaling, GO:0007267	281	38	0.000380
Cellular Ca^2+^ homeostasis, GO:0006874	94	12	0.006141
Cell fate commitment, GO:0045165	70	11	0.000924
Myofibril assembly, GO:0030239	7	3	0.003370
Molecular functions			
Signal transducer activity, GO:0004871	1678	405	0.000031
G-prot.-coupled receptor activity, GO:0004930	1653	396	0.000046
Cytokine activity, GO:0005125	232	32	0.000156
Growth factor activity, GO:0008083	190	26	0.001322
Metallopeptidase activity, GO:0004222	142	21	0.001336

Biological process and molecular function groups from gene ontology were found using Pathway Studio 7.1 from Ariadne Genomics. The results (corrected *P* value cutoff ≤0.05) were sorted based on the number of genes involved in each group. The absolute fold-change cutoff was set at 1.6

### Presence of palmitate affects the expression of genes associated with cell communication

The treatment of C2C12 myoblasts with palmitate resulted in the regulation of genes encoding cytokines (Table 5) and growth factors (Table 6). The majority of these genes were downregulated and were altered rather slightly (absolute fold-change values below 2.0) by palmitate supplementation. Ten of the differentially expressed genes overlapped both GO categories. Palmitate slightly but significantly downregulated 14 genes associated with metalloendopeptidase activity, whereas seven genes belonging to this group were upregulated (Table 7).

**Table 5:** Palmitate-regulated genes associated with cytokine activity (molecular function GO:0005125)

No.	Fold change	Gene ID	Description
Downregulated			
1.	−1.62	*IfnB1*	*Mus musculus* interferon beta 1, mRNA (NM_010510)
2.	−1.62	*CD40lg*	*M. musculus* CD40 ligand, mRNA (NM_011616)
3.	−1.64	*Gdf2*	*M. musculus* growth differentiation factor 2, mRNA (NM_019506)
4.	−1.65	*Il25*	*M. musculus* interleukin 25, mRNA (NM_080729)
5.	−1.65	*Pglyrp1*	*M. musculus* peptidoglycan recognition protein 1, mRNA (NM_009402)
6.	−1.66	*Mstn*	*M. musculus* myostatin, mRNA (NM_010834)
7.	−1.66	*Ccl22*	*M. musculus* chemokine (C-C motif) ligand 22, mRNA (NM_009137)
8.	−1.67	*Il12B*	*M. musculus* interleukin 12b, mRNA (NM_008352)
9.	−1.67	*Prl7d1*	*M. musculus* prolactin family 7, subfamily d, member 1, mRNA (NM_011120)
10.	−1.67	*Il23A*	*M. musculus* interleukin 23, alpha subunit p19, mRNA (NM_031252)
11.	−1.67	*Il1F8*	*M. musculus* interleukin 1 family, member 8, mRNA (NM_027163)
12.	−1.68	*Csf2*	*M. musculus* colony stimulating factor 2 (granulocyte-macrophage), mRNA (NM_009969)
13.	−1.68	*Cxcl3*	*M. musculus* chemokine (C-X-C motif) ligand 3, mRNA (NM_203320)
14.	−1.69	*Xcl1*	*M. musculus* chemokine (C motif) ligand 1, mRNA (NM_008510)
15.	−1.70	*Il22*	Synonym: *M. musculus* interleukin 10-related T cell-derived inducible factor beta, mRNA (NM_054079)
16.	−1.70	*Ccl26*	*M. musculus* chemokine (C-C motif) ligand 26, mRNA (NM_001013412)
17.	−1.70	*Il9*	*M. musculus* interleukin 9, mRNA (NM_008373)
18.	−1.70	*Il1f5*	*M. musculus* interleukin 1 family, member 5 (delta), transcript variant 1, mRNA (NM_001146087)
19.	−1.72	*IfnK*	*M. musculus* interferon kappa, mRNA (NM_199157)
20.	−1.72	*Tnfsf4*	*M. musculus* tumor necrosis factor (ligand) superfamily, member 4, mRNA (NM_009452)
21.	−1.74	*Ifna4*	*M. musculus* interferon alpha 4, mRNA (NM_010504)
22.	−1.78	*Bmp5*	*M. musculus* bone morphogenetic protein 5, mRNA (NM_007555)
23.	−2.01	*Il12a*	*M. musculus* interleukin 12a, transcript variant 1, mRNA (NM_001159424)
Upregulated			
24.	1.64	*InhbB*	*M. musculus* inhibin beta-B, mRNA (NM_008381)
25.	1.66	*Osm*	*M. musculus* oncostatin M, mRNA (NM_001013365)
26.	1.67	*Wnt2*	*M. musculus* wingless-related MMTV integration site 2, mRNA (NM_023653)
27.	1.76	*CD70*	*M. musculus* CD70 antigen, mRNA (NM_011617)
28.	1.99	*Tnfsf14*	*M. musculus* tumor necrosis factor (ligand) superfamily, member 14, mRNA (NM_019418)
29.	2.04	*Faslg*	*M. musculus* Fas ligand (TNF superfamily, member 6), transcript variant 1, mRNA (NM_010177)
30.	2.66	*Il17a*	*M. musculus* interleukin 17A, mRNA (NM_010552)
31.	2.69	*Bmp6*	*M. musculus* bone morphogenetic protein 6, mRNA (NM_007556)
32.	2.73	*LtB*	*M. musculus* lymphotoxin B, mRNA (NM_008518)

Mouse C2C12 myoblasts were exposed to palmitate (0.1 mmol/l) for 48 h. Data were analyzed using the Gene Spring software (Agilent, USA). The absolute fold-change cutoff was set at 1.6

*P* value ≤ 0.05

**Table 6:** Palmitate-regulated genes associated with growth factor activity (molecular function GO:0008083)

No.	Fold change	Gene ID	Description
Downregulated			
1.	−1.64	*Gdf2*	*Mus musculus* growth differentiation factor 2, mRNA (NM_019506)
2.	−1.65	*Fgf16*	*M. musculus* fibroblast growth factor 16, mRNA (NM_030614)
3.	−1.66	*Hdgfrp3*	Hepatoma-derived growth factor, related protein 3 (source: MGI Symbol; acc: MGI:1352760) (ENSMUST00000026094)
4.	−1.66	*Mstn*	*M. musculus* myostatin, mRNA (NM_010834)
5.	−1.66	*Fgf14*	*M. musculus* fibroblast growth factor 14, transcript variant 1, mRNA (NM_010201)
6.	−1.66	*Nrg3*	*M. musculus* neuregulin 3, transcript variant 1, mRNA (NM_008734)
7.	−1.67	*Il12B*	*M. musculus* interleukin 12b, mRNA (NM_008352)
8.	−1.68	*Csf2*	*M. musculus* colony stimulating factor 2 (granulocyte-macrophage), mRNA (NM_009969)
9.	−1.68	*Cxcl3*	*M. musculus* chemokine (C-X-C motif) ligand 3, mRNA (NM_203320)
10.	−1.68	*Fgf12*	*M. musculus* fibroblast growth factor 12, transcript variant 1, mRNA (NM_183064)
11.	−1.70	*Il9*	*M. musculus* interleukin 9, mRNA (NM_008373)
12.	−1.71	*Fgf3*	*M. musculus* fibroblast growth factor 3, mRNA (NM_008007)
13.	−1.71	*Tff1*	*M. musculus* trefoil factor 1, mRNA (NM_009362)
14.	−1.71	*Prom2*	*M. musculus* prominin 2, transcript variant 2, mRNA (NM_178047)
15.	−1.71	*Tdgf1*	*M. musculus* teratocarcinoma-derived growth factor 1, mRNA (NM_011562)
16.	−1.72	*Angptl3*	*M. musculus* angiopoietin-like 3, mRNA (NM_013913)
17.	−1.78	*Bmp5*	*M. musculus* bone morphogenetic protein 5, mRNA (NM_007555)
18.	−1.84	*Nrg4*	*M. musculus* neuregulin 4, mRNA (NM_032002)
19.	−2.01	*Il12a*	*M. musculus* interleukin 12a, transcript variant 1, mRNA (NM_001159424)
20.	−2.25	*Fgf4*	*M. musculus* fibroblast growth factor 4, mRNA (NM_010202)
*Upregulated*			
21.	1.63	*Rabep1*	Rabaptin, RAB GTPase binding effector protein 1 (source: MGI Symbol; acc: MGI:1860236) (ENSMUST00000100928)
22.	1.64	*InhbB*	*M. musculus* inhibin beta-B, mRNA (NM_008381)
23.	1.66	*Osm*	*M. musculus* oncostatin M, mRNA (NM_001013365)
24.	1.73	*Chia*	*M. musculus* chitinase, acidic, mRNA (NM_023186)
25.	1.81	*Fgf21*	*M. musculus* fibroblast growth factor 21, mRNA (NM_020013)
26.	2.69	*Bmp6*	*M. musculus* bone morphogenetic protein 6, mRNA (NM_007556)

Mouse C2C12 myoblasts were exposed to palmitate (0.1 mmol/l) for 48 h. Data were analyzed using the Gene Spring software (Agilent, USA). The absolute fold-change cutoff was set at 1.6

*P* value ≤ 0.05

**Table 7:** Palmitate-regulated genes associated with metalloendopeptidase activity (molecular function GO:0004222)

No.	Fold change	Gene ID	Description
Downregulated			
1.	−1.62	*Mmp8*	*Mus musculus* matrix metallopeptidase 8, mRNA (NM_008611)
2.	−1.63	*Mmp21*	*M. musculus* matrix metallopeptidase 21, mRNA (NM_152944)
3.	−1.65	*Adamts12*	*M. musculus* disintegrin-like and metallopeptidase (reprolysin type) with thrombospondin type 1 motif, 12, mRNA (NM_175501)
4.	−1.65	*Adamts19*	*M. musculus* disintegrin-like and metallopeptidase (reprolysin type) with thrombospondin type 1 motif, 19, mRNA (NM_175506)
5.	−1.66	*Fap*	*M. musculus* fibroblast activation protein, mRNA (NM_007986)
6.	−1.68	*Adam29*	*M. musculus* disintegrin and metallopeptidase domain 29, mRNA (NM_175939)
7.	−1.68	*Adamts3*	*M. musculus* disintegrin-like and metallopeptidase (reprolysin type) with thrombospondin type 1 motif, 3, transcript variant 1, mRNA (NM_177872)
8.	−1.69	*Adam1b*	*M. musculus* disintegrin and metallopeptidase domain 1b, mRNA (NM_172125)
9.	−1.69	*Mmp9*	*M. musculus* matrix metallopeptidase 9, mRNA (NM_013599)
10.	−1.70	*Gm5347*	*M. musculus* predicted gene 5347, mRNA (NM_001079931)
11.	−1.70	*Adam28*	*M. musculus* 16-day embryo head cDNA, RIKEN full-length enriched library, clone: C130072N01, product: hypothetical protein, full insert sequence (AK081736)
12.	−1.73	*Gm5346*	*M. musculus* predicted gene 5346, mRNA (NM_001025240)
13.	−1.82	*Adamdec1*	*M. musculus* ADAM-like, decysin 1, mRNA (NM_021475)
14.	−1.84	*Mme*	*M. musculus* membrane metalloendopeptidase, mRNA (NM_008604)
Upregulated			
15.	1.68	*Mmp7*	*M. musculus* matrix metallopeptidase 7, mRNA (NM_010810)
16.	1.70	*Adamts18*	*M. musculus* disintegrin-like and metallopeptidase (reprolysin type) with thrombospondin type 1 motif, 18, mRNA (NM_172466)
17.	1.79	*Adamtsl2*	*M. musculus* ADAMTS-like 2, mRNA (NM_029981)
18.	1.88	*Mmp10*	*M. musculus* matrix metallopeptidase 10, mRNA (NM_019471)
19.	2.11	*Mep1a*	*M. musculus* meprin 1 alpha, mRNA (NM_008585)
20.	2.12	*Adam2*	*M. musculus* disintegrin and metallopeptidase domain 2, mRNA (NM_009618)
21.	2.12	*Tll1*	*M. musculus* tolloid-like, mRNA (NM_009390)

Mouse C2C12 myoblasts were exposed to palmitate (0.1 mmol/l) for 48 h. Data were analyzed using the Gene Spring software (Agilent, USA). The absolute fold-change cutoff was set at 1.6

*P* value ≤ 0.05

## Discussion

The mechanisms controlling skeletal muscle mass and function as well as their potential modifications through humoral factors acting during development receive much attention. There are studies concerning the effect of different concentrations of fatty acids on the mechanisms controlling muscle cell growth and differentiation, and the reported data differ depending on experimental protocol. According to Lee et al. (2009), palmitate (0.1–10 μmol/l) had no effect on either proliferation or differentiation of C2C12 myoblasts during the 48-h exposure. At the beginning of our experiments, we used a higher concentration of palmitate, i.e., 0.5 mmol/l, which is known to cause insulin resistance in muscle cells (Alkhateeb et al. 2007; Coll et al. 2008; Bakhtiyari et al. 2010; Feng et al. 2012). However, this concentration appeared highly toxic for proliferating myoblasts (not shown); therefore, finally, we decided to treat cell cultures with fatty acid salt at concentration of 0.1 mmol/l.

In our recent study, palmitate was found to inhibit proliferation of myoblasts through a decrease in cyclin A and cyclin D1 levels and an increase in p21-cdk4 complex formation, however, it promoted cell cycle exit, myogenic differentiation, and myotube growth (Grabiec et al. 2015). On the other hand, fatty acid-induced stimulation of protein degradation via the ubiquitin-proteasome pathway and inhibition of protein synthesis through the increased phosphorylation of eukaryotic initiation factor 2 (eIF2) in skeletal muscle cells have already been described (Zhou et al. 2007). Such observations prompt the hypothesis that the effects of palmitate of skeletal myoblasts could be secondary, due to the modulation of cell response to environmental stimuli. According to our results, palmitate, a fatty acid present in blood plasma and in skeletal muscle tissue (Chavez and Summers 2003), appeared as a “pleiotropic” factor which affects expression of numerous genes important for cellular processes associated with muscle cell growth and metabolism. It should be noticed that palmitate-mediated modification of gene expression was rather slight, as the absolute fold-change values were usually below 2.0. However, the differences between control and palmitate-treated cells were observed at 48 h of exposition, and it is likely that this effect would become more evident in prolonged experiment.

The addition of palmitate affects the expression of genes involved in signal transduction pathways of hormones, cytokines, and growth factors (Table 4). The most significant process enriched in the differentially expressed genes in C2C12 myoblasts treated with palmitate was the signal transduction. The majority of genes with expression altered by palmitate treatment display the functions of a signal transducer and G-protein coupled receptors (Table 4). Decreased expression of genes related to important signaling pathways can result in impaired responsiveness of palmitate-treated myogenic cells to hormones and growth factors. In fact, a high-fat diet or acute exposure to palmitate caused insulin resistance and the relevant mechanisms have already been described (Delarue and Magnan 2007; Alkhateeb et al. 2007). The microarray analysis in our study revealed the palmitate-exerted modification of signaling pathways important for mediating effects of hormones and growth factors (Fig. 2), as well as for inflammatory response (Table 4). This is in agreement with recent reports on increased protein tyrosine phosphatase-1B (Bakhtiyari et al. 2010), decreased phosphorylation of Akt (Feng et al. 2012), or activation of NF-κB (Zhang et al. 2010) in palmitate-treated myogenic cells.

Other biological processes which were significantly associated with genes modified in palmitate-treated myoblasts included cell adhesion, cell-cell signaling, and cellular Ca^2+^ homeostasis (Table 4). Cell-matrix and cell-cell adhesions are essential for most cell types during development and normal cell function, as they regulate cell polarity, migration, and differentiation (Krauss et al. 2005; Lei et al. 2013). Genes altered in the presence of palmitate were associated with integrin, cadherin signaling, plasminogen activation, and cytoskeletal regulation (Fig. 2). Calcium homeostasis appeared to be important in the maintenance of mechanisms promoting the expression of muscle-specific proteins and myoblast fusion (Mancinelli et al. 2012). Another pathway enriched by differentially expressed genes in palmitate-treated myoblasts is Wnt signaling, which is important for early development and commitment of myogenic cells (Otto et al. 2008). Palmitate supplementation downregulated the majority of genes associated with the Wnt pathway (Fig. 2) and is in agreement with the idea presented by Du et al. (2010) concerning the inhibitory effect of maternal obesity on Wnt/beta-catenin signaling, leading to impairment of fetal myogenesis.

The addition of palmitate modifies the expression of genes encoding cytokines, growth factors, and genes related to extracellular matrix. These results raise the possibility that modification of myoblast secretome, that is, cytokine/growth factor release, by palmitate could serve as a mechanism of impact on myoblast growth. It is partially supported by the present results of selected genes’ expression of short-time vs 48-h treatment with palmitate. Only the *Angtpl4* mRNA levels were increased by palmitate within a few hours (Table 3). The inhibitory effect on *Arpc3* appeared after the 24-h exposure; moreover, the effects of fatty acid supplementation on *Tfpi* and *Usp47* after 24 (Table 3) and 48 h of treatment (Table 2) were opposite. This suggests that the effects of palmitate treatment observed after 48 h may be mainly due or may be superposed by the effects resulting from autocrine stimulation of cytokines/growth factors released by the myoblasts.

We showed a decrease in the expression of genes associated with cytokine and growth factor activity (Tables 5 and 6). This finding confirms the ability of myogenic cells to express bioactive factors, such as interleukins (Pedersen and Febbraio 2008), chemokines (Griffin et al. 2010; Ge et al. 2013; Grzelkowska-Kowalczyk et al. 2015), fibroblast growth factors (Hojman et al. 2009) and ADAMTS-like proteins (Koo et al. 2007), plasminogen activator (Francis et al. 2014), and proteoglycans (Li and Velleman 2009), involved in the regulation of muscle development and vascularization (Novak et al. 2011). It is in agreement with other recent observations reporting that saturated fatty acids are able to induce proinflammatory cytokine production (Miao et al. 2015a), through the activation of transcription factors, such as nuclear factor NF-κB, forkhead box-containing protein FOXO-1, or hypoxia-inducible factor HIF-1α (Lee et al. 2014; Miao et al. 2015b). Interestingly, high-fat diet-induced release of inflammatory cytokines can be modulated by polyunsaturated fatty acid supplementation; nevertheless, the exact roles of n3 and n6 fatty acids are not clear (Sundaram et al. 2015). Such results, once again, support the indirect mechanisms of fatty acid action. The presence of palmitate affected genes encoding cytokines and chemokines which have already been investigated in myoblasts (Griffin et al. 2010), such as Bmp6, Csf2, Xcl1, Inhbb, and Tnfsf14 (Tables 5 and 6). Moreover, in palmitate-treated myoblasts, we found modification of numerous genes encoding cytokines and chemokines, which, to our knowledge, had not been previously known to be expressed by skeletal muscle cells, i.e., Ccl22, Ccl28, Cxcl3, IL-12a, IL-12b, IL-17a, IL-22, IL-23a, and IL-25 (Table 5). The exact roles of these factors in muscle cell growth and differentiation require further research.

Some of the experimentally modified genes seem to be particularly interesting, since they are known regulators of myoblast proliferation and differentiation. Neuregulins have been described as myokines that exert relevant effects on myogenesis and the regulation of muscle metabolism (Gumà et al. 2010; Juretić et al. 2012). Oncostatin M (OSM), an IL-6 family cytokine, inhibits proliferation of myoblasts by cyclin D1 protein reduction (Kim et al. 2008) and inhibits myoblast differentiation via downregulation of MyoD and MEF2 transcription activity and stimulation of Id1 and Id2 proteins (Xiao et al. 2011). Interestingly, the presence of palmitate slightly downregulated myostatin, a negative factor for muscle cell growth and differentiation, and upregulated genes encoding health benefit myokines (Table 6). The protein products of *Fgf21* and *Chia* counteract cytokine-mediated inflammation and insulin resistance in skeletal muscle (Lee et al. 2012; Görgens et al. 2014). In our study, exposure to palmitate upregulated the expression of genes encoding these factors (Table 6), suggesting the complex alteration of myoblast microenvironment. Recently, Yang et al. (2013) found a negative effect of high palmitate (0.2–0.6 mmol/l) on C2C12 myotubes, manifested by a drop in expression of the health benefit myokines (FGF21, FnDC5, CTRP15). This apparent discrepancy with our results indicates that the effects of palmitate on myogenic cell secretome could be dose dependent. Alternatively, these differential effects could result from different susceptibility of myotubes and myoblasts to palmitate, or could indicate indirect effects, which appear during 48 h of treatment.

Another group enriched with genes modified by palmitate supplementation was associated with metalloendopeptidase activity (Table 7). We found several genes encoding ADAM, ADAMTS, and ADAMTS-like proteins, which were already described in differentiating C2C12 myocytes (Henningsen et al. 2010). Among the metallopeptidases, downregulated by palmitate in myoblasts was fibroblast activation protein (Fab), an integral membrane serine peptidase. The role of Fab in myogenic cells is unknown; however, this protein is crucial for tumor invasion and metastasis (Ding et al. 2014). It therefore cannot be excluded that the decreased expression of Fab in myoblasts affects cell abilities of matrix adhesion and migration and, in turn, cellular growth.

In conclusion, the results of our study indicate that addition of palmitate, whether acting directly or indirectly, can modify expression of genes associated with (1) myoblast responsiveness to hormones and growth factors, (2) cytokine and growth factor expression, and (3) regulation of cell-cell cell-matrix communication. Such alterations can affect myoblast growth and differentiation; however, further studies in this field are required.

## Abbreviations

Ccl: Chemokine C-C motif
CxCl: Chemokine C-X-C motif
CV: Crystal violet
DMEM: Dulbecco’s modified Eagle’s medium
DMSO: Dimethylsulfoxide
FBS: Fetal bovine serum
FFA: Free fatty acid
GO: Gene ontology
IL: Interleukin
MTT: 3-(4,5-dimethylthiazol-2-yl)-2,5-diphenyltetrazolium
NF-κB: Nuclear factor κB
PA: Palmitate
qPCR: Quantitative polymerase chain reaction
Wnt: Wingless-Int
